# Fibrosierte Hypersensitivitätspneumonie: Fokus auf pathologierelevante Aspekte der neuen klinischen Leitlinie der ATS/JRS/ALAT zur Diagnostik der Hypersensitivitätspneumonie bei Erwachsenen

**DOI:** 10.1007/s00292-020-00885-7

**Published:** 2020-12-23

**Authors:** Sabina Berezowska

**Affiliations:** Institut Universitaire de Pathologie, Centre Hospitalier Universitaire Vaudois et Université de Lausanne, Rue du Bugnon 25, 1011 Lausanne, Schweiz

**Keywords:** Exogen-allergische Alveolitis, Hypersensitivitätspneumonitis, Fibrosierende chronische interstitielle Lungenerkrankung, Idiopathische Lungenfibrose, Granulome, Exogenous allergic alveolitis, Hypersensitivity pneumonitis, Chronic fibrotic lung disease, Idiopathic interstitial Pneumonia, Granuloma

## Abstract

Die Hypersensitivitätspneumonie (Synonym: exogen-allergische Alveolitis) ist eine chronische interstitielle Pneumonie, die bei dafür anfälligen Personen als Hypersensitivitätsreaktion nach Antigenkontakt auftritt und über diese Entzündung in eine chronisch fortschreitende, letale Lungenfibrosierung münden kann. Insbesondere die fibrotische Hypersensitivitätspneumonie, die bis dato chronische Hypersensitivitätspneumonie genannt worden ist, stellt eine diagnostische Herausforderung dar. Die Abgrenzung zur idiopathischen Lungenfibrose (IPF) und kollagenoseassoziierten Lungenfibrose (CTD-ILD) kann sehr schwierig sein, wiewohl sie therapeutisch wichtig ist. Obwohl der diagnostische Goldstandard einer multidisziplinären Diskussion und damit der synoptischen Zusammenführung aller Befunde zur finalen Diagnosestellung fest etabliert ist, hat die hohe Interobservervariabilität zwischen Experten innerhalb der Kerndisziplinen (Pneumologie, Radiologie, Pathologie) als auch zwischen den multidisziplinären Teams die Notwendigkeit von Leitlinien aufgezeigt.

Im aktuellen Übersichtsartikel werden die pathologierelevante Aspekte der neuen Leitlinie der ATS/JRS/ALAT zur Diagnostik sowohl der zellulären als auch der fibrotischen Hypersensitivitätspneumonie bei Erwachsenen vorgestellt.

## Abstract (en)

Hypersensitivity pneumonia (HP), also called exogenous allergic alveolitis, is a chronic interstitial pneumonia induced by a hypersensitivity reaction to an identified or unidentified antigen in exposed and susceptible individuals that may progress to terminal lung fibrosis. The diagnosis of HP presents a diagnostic challenge. Though therapeutically important, it may be particularly difficult to differentiate fibrotic HP, historically termed chronic HP, from idiopathic pulmonary fibrosis (IPF) or interstitial lung disease associated with connective tissue diseases (CTD-ILD). Multidisciplinary discussion and thus a synoptic evaluation of all findings is firmly established as the gold standard diagnostic approach in interstitial lung diseases including HP. Nonetheless, the high interobserver variability between experts from the individual disciplines (pulmonology, radiology, and pathology) and between experienced multidisciplinary teams in assessing the diagnostic probability of HP has highlighted the need for widely accepted guidelines.

The present review summarizes pathology-relevant aspects of the new ATS/JRS/ALAT clinical practice guideline for the diagnosis of HP in adults.

Im Fokus des aktuellen Beitrags steht die fibrotische Hypersensitivitätspneumonie (HP; synonym gebraucht: Hypersensitivitätspneumonitis, exogen-allergische Alveolitis), bis dato meist chronische Hypersensitivitätspneumonie genannt, die eine wichtige Differenzialdiagnose innerhalb der Gruppe der chronischen, fibrosierenden interstitiellen Lungenerkrankungen darstellt [1]. Sie ist insbesondere auch eine wichtige Differenzialdiagnose zur idiopathischen Lungenfibrose (IPF), deren Ätiologie unklar ist [2].

Da die Fibrose auf dem Boden der HP im Gegensatz zur Lungenfibrosierung bei IPF aufgrund eines Entzündungsprozesses der Lungen auftritt, der eine Typ-III- und Typ-IV-Hypersensitivitätsreaktion nach Antigenkontakt in dafür anfälligen Menschen darstellt, ist die korrekte Diagnose auch therapeutisch wichtig. Nicht zuletzt muss zwingend die weitere Antigenexposition verhindert werden.

Leider hat sich die Diagnostik der HP vor allem im fibrosierten Stadium als problematisch herausgestellt. Wie bei allen idiopathischen, chronischen, fibrosierenden Lungenerkrankungen gilt auch für die HP, dass aufgrund sich überschneidender klinischer, radiologischer und histopathologischer Befunde die finale Krankheitsdiagnose anhand der Synopsis aller Befunde im Rahmen einer multidisziplinären Diskussion (MDD) gestellt werden sollte, um die diagnostische Sicherheit zu verbessern [1, 3]. Dabei besteht das Kernteam aus Pneumologie, Radiologie und Pathologie, und weitere Spezialisten (z. B. Rheumatologie) sollten bei Bedarf hinzugezogen werden. In einer internationalen Studie hat sich jedoch gezeigt, dass trotz optimaler multidisziplinärer Diagnostik die Interobservervariabilität bzgl. der Einschätzung der Wahrscheinlichkeit einer bestimmten Diagnose zwischen 7 sehr erfahrenen multidisziplinären Teams bei der fibrotischen HP im Vergleich mit den übrigen chronischen Lungenfibrosen am höchsten war (κ-Wert: 0,29) [4]. Dies kann auf einen fehlenden Konsensus bzgl. diagnostischer Kriterien und fehlende Leitlinien zurückgeführt werden – ein Problem, das in den letzten Jahren verstärkt thematisiert worden ist [5, 6]. Die höchste Konkordanz (κ-Wert: 0,42) war noch bei Diagnostik der fibrotischen HP anhand des isolierten pneumologischen Befundes zu beobachten [4], der den hohen Stellenwert des Auffindens einer möglichen Antigenexposition in der klinischen Diagnosestellung widerspiegelt [7].

Mit den jüngst publizierten klinischen Leitlinien der American Thoracic Society (ATS)/Japanese Respiratory Society (JRS) und Asociación Latinoamericana del Tórax (ALAT) zur klinischen Praxis der Diagnostik der HP bei Erwachsenen wurde auf den bisher fehlenden Konsensus zur Definition der HP, deren diagnostischer Kriterien und zum diagnostischen Vorgehen bei Verdacht auf HP reagiert [8].

Da der Lungenbiopsie und deren histologischer Begutachtung oft eine entscheidende Rolle bei der Diagnosefindung zukommt, wurden hier unter anderem den praktizierenden Pathologen einheitliche Kriterien an die Hand gelegt, die im Folgenden näher vorgestellt werden sollen. Es ist anzumerken, dass in den letzten Jahren bereits viele sehr gute Übersichtsarbeiten zu histologischen Veränderungen bei HP verfasst worden sind, zum Beispiel durch die Mitglieder der Pulmonary Pathology Society [5, 9, 10]. Die aktuelle Leitlinie zielt auf eine allgemeingültige Standardisierung, um dadurch eine bessere Vergleichbarkeit von Epidemiologie und Studienresultaten zu bezwecken, die sich dann in einem besseren Verständnis der Krankheit und effizienterer Prävention und Therapie widerspiegeln wird.

## Definition der Hypersensitivitätspneumonie

Die HP ist eine entzündliche und/oder fibrotische Erkrankung des Lungenparenchyms und der kleinen Luftwege, eine typischerweise immunvermittelte Erkrankung, die sich nach Antigenkontakt in dafür anfälligen Personen manifestiert, wobei das Antigen in bis zu 50 % der diagnostizierten Fälle nicht identifiziert werden kann. Bekannte Auslöser der HP sind meist organischer Natur (Mikroorganismen, Pilze, Tierproteine, wie z. B. bei der Vogelhalterlunge), aber auch nichtorganische Partikel (wie z. B. Isocyanate), Medikamente oder Metalle [11].

Eine wichtige Anpassung betrifft aktuell die Begrifflichkeit der HP, die bis dato anhand der Symptomdauer in akute, subakute und chronische HP eingeteilt wurde. Diese Einteilung war nur vage definiert und die Symptomdauer nicht konsistent mit dem klinischen Verlauf assoziiert. Da die Prognose der HP in erster Line von einer radiologisch oder histopathologisch nachweisbaren Fibrose abhängt, wurde einstimmig beschlossen, die Einteilung entsprechend anzupassen. Die HP wird nun subtypisiert in die nichtfibrotische HP (d. h. rein entzündliche/zelluläre Form) und die fibrotische HP (d. h. gemischt entzündliche und fibrotische oder rein fibrotische Form).

## Histopathologische Diagnostik

Nach Vorbild der Leitlinien für die Diagnose der UIP/IPF [12], die in diesem Heft durch die Kollegen Ludger Fink und Danny Jonigk behandelt werden, lauten die Empfehlungen zur Diagnose anhand der Histopathologie: „HP“, „wahrscheinliche HP (probable HP)“ oder „unbestimmt für HP (indeterminate for HP)“ (Tab. 1 und 2).

**Table 1:** 

HP	Wahrscheinliche HP	Unbestimmt für HP
=Typisches histopathologisches Bild der nichtfibrotischen HP. Zumindest eine Biopsielokalisation zeigt alle 3 Befunde (1) + (2) + (3) aus der folgenden Liste^a^	Zumindest eine Biopsielokalisation zeigt Befunde (1) + (2) aus der folgenden Liste^a^	Zumindest eine Biopsielokalisation zeigt: – Befund (1) oder (2) aus der folgenden Liste^a^. – Ausgewählte IIP-Muster: a) zelluläre NSIP, b) OP, c) peribronchioläre Metaplasie ohne andere Befunde, die eine fibrosierte HP suggerieren
UND: Keine Befunde, die auf eine alternative Diagnose hindeuten (siehe unten)^b^	UND: Keine Befunde, die auf eine alternative Diagnose hindeuten (siehe unten)^b^	UND: Keine Befunde, die auf eine alternative Diagnose hindeuten (siehe unten)^b^
^**a**^**Typische histopathologische Befunde der HP:**		
*1.*	*Zelluläre interstitielle Pneumonie*	
	Bronchiolozentrisch (luftwegzentriert)	
	Zelluläre NSIP	
	Lymphozyten-prädominant	
*2.*	*Zelluläre Bronchiolitis*	
	Lymphozyten-prädominant	
	±OP	
	±Schaumzellmakrophagen in terminalen Lufträumen	
*3.*	*Schlecht geformte nichtnekrotisierende Granulome*	
	Lose Epitheloidzellcluster und/oder mehrkernige Riesenzellen ± intrazytoplasmatische Einschlüsse	
	Lage im peribronchiolären Interstitium, in den terminalen Luftwegen und/oder innerhalb der polypoiden Myofibroblastenproliferate der OP	
^b^Befunde, die auf eine **alternative Diagnose** hindeuten:		
Plasmazellen > Lymphozyten		
Ausgeprägte lymphoide Hyperplasie		
Ausgeprägte gut geformte, sarkoidoseartige Granulome und/oder nekrotisierende Granulome		
Aspirat		

*HP* Hypersensitivitätspneumonie, *IIP* idiopathische interstitielle Pneumonie, *NSIP* nichtspezifische interstitielle Pneumonie, *OP* organisierende Pneumonie

^c^Ohne die sogenannte Hot-Tub-Lunge

**Table 2:** 

HP	Wahrscheinliche HP	Unbestimmt für HP
=Typisches histopathologisches Bild der fibrotischen HP. Zumindest eine Biopsielokalisation zeigt die Befunde (1) oder (2) + (3) aus der folgenden Liste^a^	Zumindest eine Biopsielokalisation zeigt Befunde (1) + (2) aus der folgenden Liste^a^	Zumindest eine Biopsielokalisation zeigt Befund (1) oder (2) aus der folgenden Liste^a^
±Zelluläre interstitielle Pneumonie ±Zelluläre Bronchiolitis ±OP	±Zelluläre interstitielle Pneumonie ±Zelluläre Bronchiolitis ±OP	±Zelluläre interstitielle Pneumonie ±Zelluläre Bronchiolitis ±OP
UND: Keine Befunde, die auf eine alternative Diagnose hindeuten (siehe unten)^b^	UND: Keine Befunde, die auf eine alternative Diagnose hindeuten (siehe unten)^b^	UND: Keine Befunde, die auf eine alternative Diagnose hindeuten (siehe unten)^b^
^**a**^**Typische histopathologische Befunde der fibrotischen HP:**		
*1.*	*Chronische fibrosierende interstitielle Pneumonie*	
	Architekturstörung, Fibroblastenfoci ± subpleurale honigwabige Fibrose	
	Fibrotische NSIP	
*2.*	*Luftweg-zentrierte Fibrose*	
	±Peribronchioläre Metaplasie	
	±Brückenbildende Fibrose	
*3.*	*Schlecht geformte, nicht-nekrotisierende Granulome*	
^b^Befunde, die auf eine **alternative Diagnose** hindeuten:		
Plasmazellen > Lymphozyten		
Ausgeprägte lymphoide Hyperplasie		
Ausgeprägte gut-geformte, Sarkoidose-artige Granulome und/oder nekrotisierende Granulome		
Aspirat		

*HP* Hypersensitivitätspneumonie, *NSIP* nichtspezifische interstitielle Pneumonie, *OP* organisierende Pneumonie

Die Lungenbiopsie spielt bei der Sicherung der Diagnose HP oft eine entscheidende Rolle. Die im Folgenden vorgestellten diagnostischen Kriterien gelten für alle Biopsiearten, basieren jedoch auf Beschreibungen in chirurgischen Keilbiopsaten. Die typischen histopathologischen Veränderungen gelten für beide Formen der HP, wobei im Fall der fibrotischen HP zusätzlich eine Fibrose vorliegt.

## Histopathologische Kriterien der nichtfibrotischen (zellulären) HP

**Figure 1:**
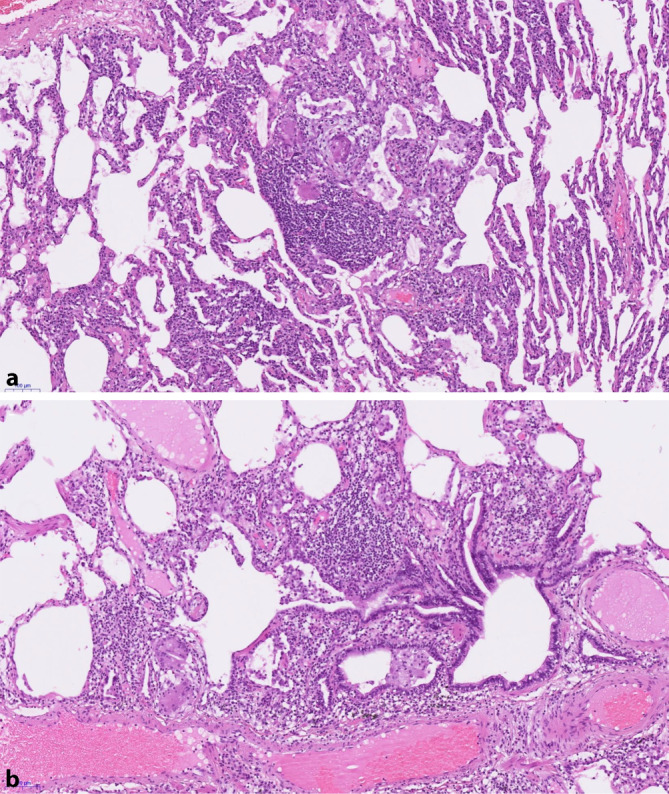

Eine sichere histologische Diagnose der HP erfordert die folgende Trias der typischen Befunde im gesamten Biopsiegut, die nicht notwendigerweise in einer einzelnen Biopsie gemeinsam vorliegen müssen (Abb. 1; Tab. 1). Zusätzlich müssen Befunde abwesend sein, die eher für eine alternative Diagnose sprechen würden.

### (1) Zelluläre interstitielle Pneumonie, die um die kleinen Luftwege akzentuiert ist (d. h. bronchiolozentrisch)

Das Entzündungsinfiltrat der HP besteht vornehmlich aus Lymphozyten, ist insgesamt jedoch polymorph und enthält daneben auch Plasmazellen und manchmal auch eosinophile Granulozyten. Lymphoide Infiltrate (insbesondere solche mit sekundären Keimzentren) finden sich nur allenfalls fokal. Eine prominente plasmazelluläre Entzündungskomponente oder eine peribronchioläre follikuläre Hyperplasie sprechen eher für eine Kollagenose-assoziierte interstitielle Lungenerkrankung (CTD-ILD) [13] oder Formen der Immundefizienz. Zum Beispiel sollte man bei einer peribronchiolären follikulären Hyperplasie mit Nachweis von Granulomen an eine granulomatöse-lymphozytäre ILD (GL-ILD) denken, die als Lungenbeteiligung bei variablem Immundefektsyndrom („common variable immunodeficiency disorder“, CVID) vorkommt, jedoch nicht spezifisch ist [14]. Das lymphoide Infiltrat ist hier deutlich stärker ausgeprägt als bei der HP und lässt eher an die Differenzialdiagnose eines Lymphoms denken.

### (2) Zelluläre chronische Bronchiolitis

Die für eine HP charakteristische chronische Bronchiolitis steht in einem Kontinuum mit der bronchiolozentrischen chronischen Pneumonie und beinhaltet die Verbreiterung des peribronchiolären Interstitiums durch das oben beschriebene chronische Entzündungsinfiltrat ohne oder mit nur fokalen Lymphfollikeln ohne sekundäre Keimzentren. Die betroffenen Bronchiolen können Foci einer assoziierten, bronchiolozentrischen organisierenden Pneumonie zeigen, d. h. im Lumen der Alveolen und terminalen Bronchiolen liegende polypoide Myofibroblastenproliferate. Als Zeichen einer Mikroobstruktion und Dysfunktion der kleinen Luftwege können in den peribronchiolären Alveolarräumen zahlreiche schaumzellige Makrophagen nachweisbar sein.

Die chronische Bronchiolitis kann auch mit einer peribronchiolären Metaplasie einhergehen. Dabei zeigt sich eine Verbreiterung des peribronchiolären Interstitiums und der angrenzenden, durch metaplastisches Bronchialepithel ausgekleideten Alveolarsepten durch eine sehr geringe Fibrose, die die Lungenarchitektur nicht beeinträchtigt. Auch hier können Foci einer organisierenden Pneumonie zu finden sein.

### (3) Bestimmtes Muster einer granulomatösen Entzündung

Der diagnostische Wert der granulomatösen Entzündung ist stark abhängig von der Art der Granulome. Die HP-typischen Granulome sind klein und schlecht geformt und beinhalten kleine, lose, unscharf umschriebene Gruppen von epitheloidzelligen Makrophagen und mehrkernigen Riesenzellen, die vor allem im peribronchiolären Interstitium anzutreffen sind. Häufig finden sich auch isoliert liegende mehrkernige Riesenzellen, die häufig intrazytoplasmatische Einschlüsse aufweisen, wie z. B. Schaumann-Körperchen (rund-ovale, lamelläre Verkalkungen), Asteroidkörperchen (sternförmige Einschlüsse) oder Cholesterinkristalllücken. Die Granulome und mehrkernigen Riesenzellen können auch in die peribronchiolären Alveolarräume übertreten und dort auch innerhalb der intraalveolären Myofibroblastenproliferate der organisierenden Pneumonie zu finden sein. Sie sollten jedoch auch interstitiell vorliegen. Gut geformte Granulome wie bei Sarkoidose und Infekten sind bei der HP selten und sollten nicht überwiegen. Eine weitere wichtige Differenzialdiagnose ist die Aspirationspneumonie, deren Granulome aber meist nur intraalveolär liegen oder assoziiert sind an eine organisierende Pneumonie. Die Granulome bei Aspiration sind zudem häufig gut geformt und zeigen Mikroabszesse, mit kleinfokalen Nekrosezentren und assoziiert (teils nur einzelnen) neutrophilen Granulozyten. Eine Suche nach Aspirat ist in diesem Fall angezeigt [15].

Die Hot-Tub-Lunge ist eine mit *Mycobacterium-avium*-Komplex assoziierte diffuse Lungenerkrankung mit klinischen und radiologischen Befunden, die mit dem klassischen Subtyp der HP überlappen. Es finden sich typischerweise gut geformte Granulome mit oder ohne zentrale Nekrose, die auf die Lumina der distalen Bronchiolen beschränkt sind.

Grundsätzlich sollte bei Nachweis von nicht mit Anthrakose assoziierten Granulomen aufgrund der großen klinischen Konsequenz eine Abklärung des Befundes hinsichtlich einer infektiösen Genese mithilfe von Spezialfärbungen erfolgen (Ziehl-Neelsen, Grocott-Versilberung) [15]. Eine zusätzliche molekulare Untersuchung zum Nachweis mykobakterienspezifischer DNA kann unter Berücksichtigung der klinischen Situation notwendig sein.

Die Diagnose einer wahrscheinlichen HP („probable HP“) trifft auf Fälle zu, in denen nur einige der 3 für HP typischen Befunde vorhanden sind. Sie erfordert eine lymphozytenreiche, bronchiolozentrische interstitielle Pneumonie und eine assoziierte Bronchiolitis, aber ohne die granulomatöse Entzündung, die für eine klassische HP charakteristisch ist.

Unbestimmt für HP („indeterminate for HP“) bezieht sich auf Fälle mit entweder zellulärer bronchiolozentrischer interstitieller Pneumonie oder einer ansonsten nicht erklärbaren zellulären chronischen Bronchiolitis, aber wiederum ohne die HP-typische granulomatöse Komponente.

## Histopathologische Kriterien der fibrotischen HP

**Figure 2:**
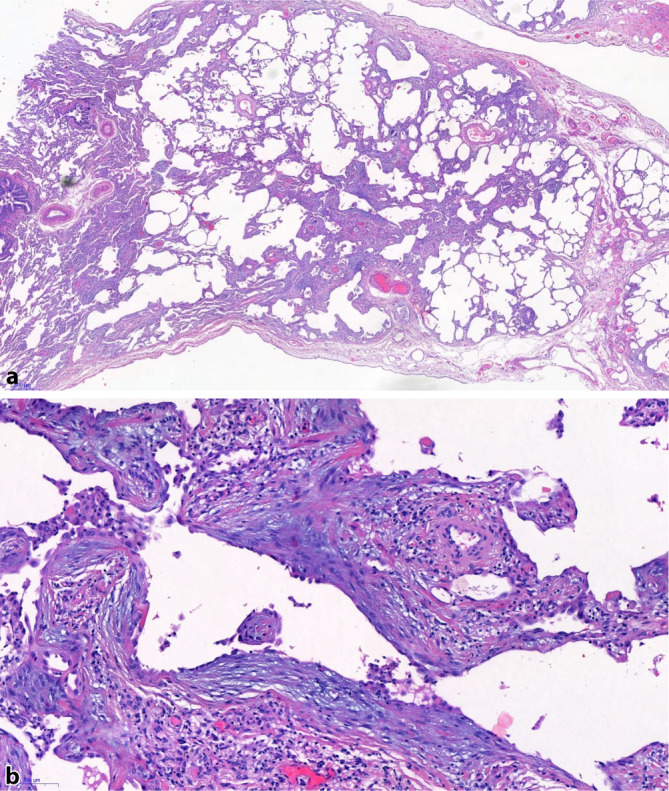

Die fibrotische HP unterscheidet sich von der nichtfibrotischen HP dadurch, dass die zugrunde liegende chronische interstitielle Pneumonie und/oder Bronchiolitis durch eine Lungenfibrose verkompliziert wird. Die typischen histologischen Charakteristika sind eine subpleurale und zentriazinäre Lokalisation der Fibrose mit oder ohne brückenbildender Fibrose zwischen zentriazinären Regionen untereinander oder zwischen subpleuralen und zentriazinären Regionen (Abb. 2).

Das Muster der fibrotischen interstitiellen Pneumonie kann Befunde beinhalten, die mit dem Muster einer gewöhnlichen interstitiellen Pneumonie („usual interstitial pneumonia“, UIP) überlappen, z. B. fleckförmige kollagenreiche Fibrose, Fibroblastenfoci und eine subpleural betonte, honigwabenartige Fibrose. Manche Experten haben den Begriff „UIP-artig“ verwendet, um die Aufmerksamkeit auf die histologische Überschneidung mit dem UIP-Muster zu lenken, welches häufig differenzialdiagnostische Probleme bietet. Da der Begriff „UIP-artig“ potenziell verwirrend sein kann, wird seine Verwendung in den neuen Leitlinien ausdrücklich nicht empfohlen, obwohl das Problem der differenzialdiagnostischen Abgrenzung zwischen fibrotischer HP und UIP explizit anerkannt wird.

In anderen Fällen hat die interstitielle Pneumonie eine eher uniforme und diffuse Ausprägung ohne honigwabenartigen Lungenumbau und kann das Muster einer fibrotischen nichtspezifischen interstitiellen Pneumonie (NSIP) imitieren („NSIP-artig“). Die peribronchioläre Fibrose zeigt sich typischerweise als eine peribronchioläre Metaplasie mit Fibrose, ein Befund mit signifikanter histologischer Überschneidung zu einer sogenannten interstitiellen bronchiolozentrischen Fibrose/atemwegszentrischen Fibrose [16, 17]. Weder die peribronchioläre Metaplasie noch die bronchiolozentrische Fibrose sind spezifisch für die HP und sind daher *per se* nicht diagnostisch für eine HP. Sie sind jedoch häufiger und stärker ausgeprägt in Patienten mit fibrotischer HP im Vergleich zu Patienten mit anderen fibrotischen interstitiellen Pneumonien. Im Fall eines UIP-Musters ist die peribronchioläre Metaplasie oder bronchiolozentrische Fibrose jedoch nicht mit einer besseren Prognose assoziiert [18].

Die Unterscheidung einer fibrotischen HP von anderen fibrotischen interstitiellen Pneumonien basiert auf dem Nachweis einer zentriazinären Fibrose und dem Vorliegen der oben beschriebenen, für eine nichtfibrotische HP typischen Befunde, die in weniger fibrosierten bzw. nichtfibrosierten Lungenarealen vorliegen. Dies erfordert die Biopsieentnahme aus mehreren Lokalisationen. Die Befunde können hier heterogen ausgeprägt sein. In einer Biopsie könnte z. B. ein Muster vorliegen, das nicht zu unterscheiden ist von einer UIP bei IPF, während andere Biopsien die Charakteristika einer nichtfibrotischen HP zeigen können, einschließlich solcher, die eher als „wahrscheinliche HP“ oder „unbestimmt für HP“ charakterisiert werden können. Diese diagnostische Diskordanz zwischen einzelnen Regionen ist analog zu der histopathologischen Variabilität in chirurgischen Biopsien von Patienten mit IPF, die neben Regionen mit UIP-Muster auch häufig Biopsien mit lediglich NSIP-artigen Veränderungen aufweisen [19].

Das Vorliegen einer Fibrose muss im Befund dokumentiert werden, da diese einen negativen Prognosefaktor darstellt.

## Diagnostische Kriterien der finalen, integrativen Diagnose einer HP

Die finale Diagnose einer HP bedingt die Integration der Befunde aller Disziplinen, die optimal im Rahmen der MDD erfolgt. Die fibrotische HP sollte als Differenzialdiagnose bei allen Patienten mit fibrotischer ILD in Erwägung gezogen werden.

Trotz methodischer Einschränkungen vieler bisheriger Studien haben sich Schlüsselbefunde für die Diagnose einer HP herauskristallisiert, die in die diagnostischen Kriterien der vorliegenden Leitlinien integriert worden sind. Darunter zählen (a) die Identifikation einer Antigenexposition (Anamnese oder Serum-IgG-Testung auf spezifische Antigene oder spezifischer Inhalationsprovokationstest), (b) eine typische radiologische Bildgebung und (c) typische histopathologische Befunde, wie sie für die nichtfibrotische und die fibrotische HP in den vorherigen Abschnitten detailliert beschrieben sind, sowie eine Lymphozytose von 30 % in der bronchoalveolären Lavage (BAL). Die einzelnen Befunde für sich genommen sind weder diagnostisch noch zwingend notwendig. Kombinationen von Befunden schlagen sich in unterschiedlicher diagnostischer Sicherheit für das Vorliegen einer HP nieder, die als Konsensus des Komitees erarbeitet worden sind und in Tab. 3 aufgelistet werden. Dabei sollte bedacht werden, dass in der Diagnostik interstitieller Lungenerkrankungen eine sichere Diagnose unter Verwendung der am wenigsten invasiven Methodik angestrebt werden sollte.

**Table 3:** 

	HRCT typisch für HP		HRCT vereinbar mit HP		HRCT unbestimmt für HP	
Exposition und/oder Serum-IgG	+	−	+	−	+	−
Keine BAL oder BAL: keine Lymphozytose *und* entweder keine Histologie oder unklarer histopathologischer Befund	Mäßige Konfidenz	Niedrige Konfidenz	Niedrige Konfidenz	HP nicht ausgeschlossen	HP nicht ausgeschlossen	HP nicht ausgeschlossen
**BAL:** Lymphozytose ohne Histologie	Hohe Konfidenz	Mäßige Konfidenz	Mäßige Konfidenz	Niedrige Konfidenz	Niedrige Konfidenz	HP nicht ausgeschlossen
**BAL:** Lymphozytose + unklarer histopathologischer Befund	Definitiv	Hohe Konfidenz	Mäßige Konfidenz	Mäßige Konfidenz	Niedrige Konfidenz	HP nicht ausgeschlossen
**Histologie:** wahrscheinliche HP	Definitiv	Hohe Konfidenz	Hohe Konfidenz	Mäßige Konfidenz	Mäßige Konfidenz	Niedrige Konfidenz
**Histologie:** typische HP	Definitiv	Definitiv	Definitiv	Definitiv	Definitiv	Hohe Konfidenz^a^

Die diagnostische Sicherheit wird in 3 Konfidenzniveaus wiedergegeben: definitiv bei ≥90 % Konfidenz; hoch bei 80–89 % Konfidenz; mäßig bei 70–79 % Konfidenz und niedrig bei 51–69 % Konfidenz. Fälle aller Konfidenzniveaus sollten interdisziplinär besprochen werden.

*HRCT* hochauflösende Computertomographie, *HP* Hypersensitivitätspneumonie, *BAL* bronchoalveoläre Lavage

^a^Das Konfidenzniveau kann auf „definitiv“ angehoben werden, wenn die histopathologische Diagnose auch nach Reevaluation im Kontext aller zusätzlichen klinischen/radiologischen Befunde weiterhin bestehen bleibt oder durch eine Experten-Zweitmeinung bestätigt wird.

Weibliches Geschlecht, ein charakteristischer Auskultationsbefund, fehlende Raucheranamnese und eine obstruktive oder gemischt restriktiv-obstruktive Physiologie haben eine deutlich geringere Voraussagekraft für das Vorliegen einer HP und werden in den diagnostischen Kriterien der Leitlinie nicht berücksichtigt.

## Empfehlungen zur Durchführung diagnostischer Interventionen

Anhand des GRADE-Systems zur Bewertung der Qualität der Evidenz und zur Einstufung der Empfehlungsstärke im Rahmen von Leitlinien wurden vom Leitlinien-Komitee Empfehlungen zur Durchführung von BAL und Biopsien in Patienten mit neu diagnostizierter ILD und Differenzialdiagnose einer HP anhand radiologischer und/oder klinischer Befunde erarbeitet, die im Folgenden kurz zusammengefasst werden.

1. In Patienten mit Differenzialdiagnose einer nichtfibrotischen oder fibrotischen HP soll eine Abklärung einer möglichen Lymphozytose in der BAL erfolgen (Schwellenwert: 30 % Lymphozyten).
2. Im Fall der Differenzialdiagnose einer nichtfibrotischen HP sollten transbronchiale Zangenbiopsien durchgeführt werden. Das Komitee gibt keine Empfehlungen für oder gegen transbronchiale Kryobiopsien. Eine chirurgische Keilbiopsie sollte nur dann erfolgen, wenn keine Diagnose unter Zuhilfenahme weniger invasiver diagnostischer Methodik möglich war und idealerweise die Indikationsstellung nach MDD erfolgte.
3. Im Fall der Differenzialdiagnose einer fibrotischen HP gibt das Komitee keine Empfehlungen für oder gegen transbronchiale Zangenbiopsien. Kryobiopsien oder chirurgische Keilbiopsien sind empfohlen, wobei auch hier die chirurgische Keilbiopsie Fällen vorbehalten ist, bei denen anhand der übrigen Befunde keine Diagnosefindung möglich war. Die Indikationsstellung sollte im Rahmen einer MDD erfolgen.

## Fazit für die Praxis

- Die Hypersensitivitätspneumonie (HP) muss als Differenzialdiagnose jeder neu diagnostizierten interstitiellen Lungenerkrankung bedacht werden.
- Die HP wird unterteilt in die nichtfibrotische und fibrotische (ehemals: chronische) HP, da die Lungenfibrosierung prognoseentscheidend ist. Falls vorhanden sollte daher eine Fibrose im Befund angegeben werden.
- Typische Befunde einer HP sind eine Lymphozytose in der bronchoalveolären Lavage (BAL) (>30 %) sowie die typische Trias aus lymphozytenprädominanter interstitieller Pneumonie und Bronchiolitis und schlecht geformten, nichtnekrotisierenden Granulomen. Sie erleichtern bei Vorhandensein die Diagnose im Fall einer fibrotischen HP.
- Granulome sollten auf eine infektiöse Genese hin abgeklärt werden (Ziehl-Neelsen-Färbung, Grocott-Versilberung, evtl. molekulare Untersuchung).
- Differenzialdiagnosen einer fibrotischen HP sind vor allem die idiopathische Lungenfibrose (IPF) mit dem histologischen Bild einer gewöhnlichen interstitiellen Pneumonie (UIP) sowie andere idiopathische interstitielle Pneumonien, wie z. B. Kollagenose-assoziierte interstitielle Lungenerkrankung (CTD-ILD) oder mit Medikamenteneinnahme assoziierte ILD.
- Der Goldstandard für die finale Diagnosestellung einer HP stellt die synoptische Evaluation aller Befunde in der multidisziplinären Besprechung dar.
